# Behavioural change interventions for sustained trachoma elimination

**DOI:** 10.2471/BLT.18.212514

**Published:** 2018-09-10

**Authors:** Sarity Dodson, Anne Heggen, Anthony W Solomon, Virginia Sarah, Geordie Woods, Leah Wohlgemuth

**Affiliations:** a The Fred Hollows Foundation, 52 Barry St Carlton VIC 3053, Sydney, Australia.; b The Fred Hollows Foundation, Addis Ababa, Ethiopia.; c Department of Control of Neglected Tropical Diseases, World Health Organization, Geneva, Switzerland.; d The Fred Hollows Foundation, London, England.; e Sightsavers, New Orleans, United States of America.; f Sightsavers, London, England.

Trachoma is the leading infectious cause of blindness. Significant progress has been made towards the elimination of trachoma as a public health problem since the launch, in 1996, of the Alliance for the Global Elimination of Trachoma by 2020, and the endorsement of the alliance’s goal by the Member States of the World Health Organization (WHO) in 1998.^1^ Elimination is within reach if the global health community maintains focus, continues to innovate and collaborate and secures the necessary resources.

Strong international collaboration, high-quality prevalence data,^2^ the evidence-based Surgery, Antibiotics, Facial Cleanliness, Environmental Improvement (SAFE) strategy endorsed by WHO,^3^ Pfizer’s azithromycin donation scheme,^4^ significant donor support and strong political will have enabled a tremendous programmatic scale-up towards the elimination of trachoma. In 2017 alone, 84 million people received antibiotics for trachoma and more than 231 000 people received trichiasis treatment.^5^

The effects of these efforts are now being observed. Between 2007 and 2018, the number of people at risk of trachoma-related blindness dropped from 1244 million^6^ to 158 million.^5^ In 2018, Nepal and Ghana became the sixth and seventh countries validated by WHO as having eliminated trachoma as a public health problem.

## Behaviour change interventions

The SAFE strategy guides trachoma programming. Most of the strategy’s impact has been attributed to successful implementation of the surgery and antibiotics components, which are each supported by compelling evidence of effect. While numerous studies on the facial cleanliness and environmental improvement components have been reported, robust prospective evidence of their impact on trachoma prevalence is lacking: systematic reviews have been unable to demonstrate efficacy of any given approach.^7^^,^^8^

Facial cleanliness and environmental improvement are nevertheless often said to be critical to sustained trachoma elimination. They are closely linked to the fundamental human rights to water, sanitation and hygiene. Specific targets have been established for water, sanitation and hygiene within the sustainable development goals on water and sanitation and on education, driven by considerations beyond the importance of these targets to vision and health. Efforts towards universal access to water, sanitation and hygiene are ongoing. Meanwhile, trachoma programmes must seek to implement targeted, evidence-based interventions that will sustainably decrease transmission of trachoma’s causative organism, ocular *Chlamydia trachomatis*.

A range of environmental factors and behaviours associate with more intense *C. trachomatis* transmission, including poor water and sanitation access, high-fly densities, suboptimal hygiene practices (particularly face washing) and overcrowded sleeping quarters. Interlinked intervention strategies that target a range of behavioural, sociocultural and environmental factors may be needed, each considering existing resources, programmes and norms. This complexity presents challenges for programme design, monitoring and evidence synthesis and sharing, and has slowed identification of the active ingredients of effective facial cleanliness and environmental improvement interventions.

Facial cleanliness and environmental improvement studies originate from many programmes delivered in heterogeneous settings, and include intervention components described in diverse ways. Resulting measurement and reporting limitations probably prevent the results of facial cleanliness and environmental improvement interventions from being observed at scale.

A further concern is the complexity of the strategies employed and the limited reference to theory as a basis for programme design. A recent review^9^ noted the predominance, in facial cleanliness and environmental improvement programming, of awareness-raising activities and delivery of water, sanitation and hygiene equipment. Few programmes target more direct determinants of behaviour or employ approaches to ensure maintenance of behaviour change. Only a quarter of documents reviewed cited behavioural frameworks as a basis for programme design.^9^

## What should be done?

In the absence of clear evidence for facial cleanliness and environmental improvement programming, the broader health promotion and behaviour change literature can provide guidance. Several frameworks are potentially helpful and support three key decisions. First, what processes ensure effective, contextualized and participatory programme design? Second, what types of intervention should be developed? Third, how should specific programme components be selected and labelled for study and discussion?

There are several useful models of design process.^10^^,^^11^ The Ophelia Approach, for example, summarizes steps, from need recognition to solution identification across three phases of activity: situational analysis; intervention co-design; and implementation, evaluation and ongoing improvement. The cyclical flow of the approach seeks to help uncover what works, in what circumstances and how.^11^ This is consistent with the International Coalition for Trachoma Control programming guidance on facial cleanliness and environmental improvement,^12^ which highlights the importance of collaborative local programme development informed by sound understanding of the local environment and community, and ongoing monitoring and evaluation to refine intervention strategies.

A second class of frameworks guides the determination of the kind of intervention required. It is critical that theories of behaviour and its determinants underpin facial cleanliness and environmental improvement programmes. A key challenge here is the number and diversity of theories offered by a range of disciplines. Recently, however, synthesized theoretical frameworks have been developed. One such framework is the Behaviour Change Wheel, which describes the essential conditions (capability, opportunity, motivation) required for a given behaviour, the different policy intervention categories, and the various intervention functions (education, modelling and coercion).^13^

Experience suggests that for health-related behaviour change to take place and be sustained, a multilevel, evolving combination of interventions may be required over an extended period. Successful programmes to address smoking, for example, have used advertising bans, cigarette taxes, health warnings on packages, legislation restricting smoking in public places and implementation of smoking cessation community support programmes.

Such frameworks can help to ensure that interventions are optimized to achieve and sustain the desired change, by encouraging intervention designers to draw from theory and respond to the complexity of the underlying issues. For facial cleanliness and environmental improvement, these frameworks can assist programme designers to go beyond interventions focused on raising awareness of trachomatous visual impairment, building latrines and installing water points. In Ethiopia, for example, a strategic framework for facial cleanliness and environmental improvement programming has been developed. This framework adopts a comprehensive, multilevel approach and articulates three core programme elements. First, integration of neglected tropical disease and water, sanitation and hygiene programmes, whereby trachoma-specific messaging, interventions and indicators are mainstreamed across existing water, sanitation and hygiene programmes. Second, participatory approaches to deliver school-based, women-focused, community-level and mass media campaigns targeting water, sanitation and hygiene-related behaviour change. Mothers, in particular, are targeted, as they are the primary caregivers for children and infection is predominantly carried by young children. Third, institutional capacity building and infrastructure investment to improve water, sanitation and hygiene access.

A critical limitation of current facial cleanliness and environmental improvement programme design, evaluation and reporting is the lack of specificity and consistency in programme descriptions. For example, what are the critical – and what are the potentially redundant – ingredients of community-led total sanitation and hygiene^14^ or existing school-based programmes? What other techniques are needed to supplement the programmes currently in place? Without an agreed terminology to describe the existing strategies, consistent measurement of effect is challenging and the lessons generated by evaluations and from exchanges with water, sanitation and hygiene partners cannot contribute to the development of a sound evidence base for preventing blindness from trachoma. In this case, replication and scale-up of interventions cannot be reliably undertaken.

The Behaviour Change Technique Taxonomy project (2010–2013) involved developing a comprehensive agreed list of behaviour change techniques: “the smallest components of behaviour change interventions that on their own in favourable circumstances can bring about change.”^15^ This taxonomy can be tailored to particular behavioural targets, such as face washing, and used as a foundation for trachoma intervention design, evaluation and evidence synthesis. Building on that project, the Human Behaviour Change Project^16^ seeks to develop a comprehensive model of human behaviour.

## A roadmap

To reduce trachomatous blindness through behaviour change interventions and understand what works, in what circumstances and how, the global trachoma community must draw more effectively upon available behaviour change theories. We must then develop interventions, in partnership with communities and local actors, based on the consideration of the behavioural targets, context, and implementation constraints and opportunities. Frameworks exist to guide programme design, implementation and evaluation.

We must also move towards the use of a common terminology for facial cleanliness and environmental improvement interventions, greater specificity in the global trachoma community reporting of intervention components, and use of a common set of indicators to measure programme outcomes. A relatively simple exercise that could transform the field of facial cleanliness and environmental improvement programming to one in which data generated across different programmes could be compared and integrated, is the construction of a behaviour change technique taxonomy specific to trachoma elimination. The taxonomy’s structure would need to consider behaviour change at the level of the individual, the household, the school and the community. Guidance is also needed to support programme developers to consider appropriate techniques relating to both initiation and maintenance of behaviour change, to analyse how techniques might be effectively combined and to identify appropriate process and outcome indicators.

The facial cleanliness and environmental improvement components of the SAFE strategy are probably very important for preventing vision loss from trachoma. More work is needed to demonstrate that importance and to facilitate the scale-up of effective interventions.
